# St. Louis Medical Society

**Published:** 1877-08

**Authors:** 


### ST. LOUIS MEDICAL SOCIETY.

April 28th, 1877.

The President, Dr. Scott, called the Society to order.

Dr. Kennard reported the following case: A negro, aged about 32, occupation of waiter, of temperate habits, complained of indisposition the day before he came under observation. He seemed to be under the influence of an opiate, but it was learned that no drug had been taken. He was conscious, but could neither move nor speak. Pulse 72; the skin cool and moist; the pupils responded. He seemed to suffer from pain in the throat. External applications were ordered to the throat, and quinine and purgatives given internally. On inquiry it was learned that he suffered from a similar attack about a year ago.

*Biliary Calculi.*—Dr. Newman reported the case.

Last fall was requested to see a gentleman who suffered from pulmonary hemorrhage. The family history was bad, and as winter was approaching he was sent to the South, and the patient spent the winter in Texas.

He reported improvement until about three weeks before his departure for home, when he was seized with pain in the right side and pit of the stomach. Several physicians were consulted, one of whom diagnosed hydatids of the liver. He returned home, and was seen shortly afterwards when he was suffering from another attack (there was an intermission between the attacks); besides the pain there was considerable jaundice. The stools were ordered preserved, but by some mistake they were not seen. Several days afterwards a recurrence took place, and in the preserved stool a calculus was found; subsequently others were detected. His general condition was very precarious. He was put upon alkalies, and in a few days he passed large quantities of broken up calculi. Was not prepared to say whether the treatment effected this or not.

In answer to a question from Dr. Bryson, as to what salts of potassium he had employed, and their doses, Dr. Newman said he had given potass. carbon. in grs. x, and potass. acet. in grs. xx doses, together with bismuth and pepsin. The last two drugs were administered with a view to ameliorate the condition of his bowels and stomach, for everything which passed his bowels seemed undigested. The patient has had no attack since three weeks, and for the last two weeks he had passed no debris.

---

May 5th, 1877.

In the absence of the President, the Vice-President, Dr. Briggs called the Society to order.

Dr. Hodgen reported three cases of aneurism. (Published in the June number of this journal.) After reading the histories of the cases, he remarked that a difference of opinion had existed among the gentlemen who examined the third case reported, that of aneurism of the ascending portion of the arch of the aorta; some considered it an aneurism, others, amongst these himself, had diagnosed malignant enchondroma of the first rib.

Dr. Prewitt said that he had examined the case, and from the elasticity of the tumor, the impulse felt, and the marked emphasis of the heart sounds, he had diagnosed aneurism of the innominata.

Dr. Glasgow: When I first saw the case there was a fluctuating, elastic tumor, dull on percussion, and situated under the sternal end of the clavicle and first rib. Two sounds were discovered in it; later a weak bruit with the first sound and a second sound, both synchronous with the heart sounds, but more intense, were heard. There was no difference in the pulsation of the carotids; towards the end a difference in the radial pulse was observed. The larynx was congested, causing huskiness of the voice.

In answer to the question as to how he explained the origin of the sounds in the sac, he said: The sounds are heard in all aneurisms; they are produced by regurgitation in the sac.

After the recess which the Society took to enable the gentlemen to examine the specimens, Dr. Hodgen remarked that during the intermission the question had been put to him why he considered this case to be one of malignant enchondroma. In answer he said:

Within a short time I had seen two cases of malignant enchondroma, involving the sternum and costal cartilages; the specimen from one I exibited to the Society some months ago, the other is yet under observation. Now we are a little apt to run in grooves. I had not yet gotten out of the enchondroma groove. This, of course, left my mind in a favorable condition to look for and find a tumor of that character. The original site of the tumor, almost directly opposite the cartilage of the first rib, led me to differentiate between a disease of that rib and an aneurism of the innominata artery.

I did not find the radial pulse of the right side behind that of the left, nor did I uniformly find the right radial pulse less full and strong than the left; and, I reasoned, if this be an aneurism of the innominata, the pulse would be full and the flow retarded. The first and second sounds of the heart were more distinctly heard at the tumor than directly over the heart, which was explained by assuming a bettor conductor than the blood in the aorta and aneurismal sac. The growth of the tumor was most rapid in the direction of the sternum and ribs, where there would have been most resistance to the pressure of an aneurism, and the growth was least rapid above the clavicle, where the loose connective tissue would offer little resistance. The clavicle, sternum, and ribs were involved in the tumor, and to the touch seemed thickened as they approached the softer and more prominent points, and at the extreme margin of the bones the thin and easily yielding parts of bone could be felt to break down under pressure.

Besides, there were hard points scattered throughout the tumor, which indicated a malignant growth rather than an aneurism. The recurrent laryngeal nerve gave no evidence of disturbance through disturbance of the movement of the vocal cords. At several points about the margin of the tumor the minute vessels of the skin were dilated, as though the extreme vascular condition of the tumor was involved in the skin itself. Little of the severe pain that ordinarily results from pressure of an aneurism of the innominata on the axillary plexus was present. No damming back of the venous blood, as would have resulted from the pressure of an aneurism of the innominata on the veins returning the blood from the subclavian veins, was observed.

Though in error in my diagnosis, yet there were several reasons for the opinion given, although they were all fallacious. The symptoms of aneurism, as present in this case, were only such as are present in tumors not aneurismal. The site, though that most frequent in aneurisms, is one that admits of the presence of other tumors. The pulsation is found in many tumors not aneurismal; the lateral enlargement on cardiac contraction is not peculiar to aneurisms, and finally the refilling after pressure had reduced the size of the tumor, also exists in vascular malignant tumors of bone.

While I could not, if I desired, recall my error, I cannot believe, even after a post-mortem has shown how worthless my reasons are in this case, that they are quite as valuable as any that can be presented for the opinion in the earlier stage of the disease, that it was an aneurism. Some will recall a case in which age, tumor, pulsation, expansion, thrill and murmur, all existed from hydræmia.

Dr. Kennard said that hydræmia is often mistaken for aneurism. A lady came under observation who was extremely emaciated, and from the symptoms observed, aneurism of the abdominal aorta was suspected. The patient was treated for ulcers of the stomach, from which she suffered, and made a complete recovery.

---

May 12th, 1877.

*Deformed Fœtus.*—Dr. Youngblood exhibited a fœtal monstrosity about four months old. The deformity consisted rather in the absence of parts than in a peculiar development. The fœtus is armless, the femurs are absent, the knees are attached to the pelvis, there are but three toes on each foot.

Dr. Hodgen presented two specimens of cancer, one of the penis and the other of the stomach; as also a specimen illustrating follicular degeneration of the large intestine. The case of cancer of the penis was Dr. Johnston's patient, who gave the following history: About ten weeks ago the patient noticed a slight discharge from his penis, as also a small growth at the frenum. He consulted a physician, who treated him for syphilis. The growth enlarged until the whole penis and part of the scrotum was involved. There was no glandular enlargement. The penis was amputated, and the patient is now under treatment.

The second case—that of carcinoma of the stomach—was seen but once before death. The patient was between fifty-five and sixty years of age, very much emaciated, and was unable to retain anything on the stomach unless it was very slowly swallowed. The food seemed to accumulate in the œsophagus, and though retained there for a short time, was afterwards vomited. A partial autopsy was allowed. There was great thickening at the cardiac and pyloric ends of the stomach. The peritoneum and intestines were studded with cancerous deposits. The omentum was very much contracted, the kidneys and liver were normal.

*Case third.*—The patient had suffered for a number of years from dysentery, but had kept up strength pretty well. About five months ago she was again attacked with dysentery; a large quantity of mucous and blood was discharged; there was great tenesmus. A tumor appeared in the left iliac region, apparently six or eight inches in length; it varied in size and position. Intussusception of the sigmoid flexure was suspected. At the post-mortem no trace of peritonitis was discovered, except on the brim of the pelvis on the right side. An abscess had formerly occupied the ventes ilii. There were old adhesions between the colon and the margin of the pelvis. The piece of intestine shown had its follicles very much enlarged, and the mucous and sub-mucous tissues partly destroyed.

*Poisoning by the Self-inhalation of Chloroform.*—Dr. Briggs reported the case, and remarked that the habit of inhaling chloroform is prevalent amongst females to an alarming extent. Dr. Boislinere made some remarks at length on the unauthorized sale of chloroform and other dangerous drugs by apothecaries.

---

May 19, 1877.

The Society met as usual, the Vice-President, Dr. Briggs, presiding.

*Diphtheritic Membrane.*—Dr. Mudd presented a complete cast of the larynx and bronchial tubes as far as the third division which was expelled through the opening made in the trachea during tracheotomy. He gave this account of the case: The patient, a girl, nine years of age, had taken a cold and suffered considerably from coryza. Two days previous to her coming under the observation of Dr. Mudd, the attending physician, had observed diptheritic patches. An examination revealed considerable laryngeal obstruction and laborious breathing; the patient was comatose; mucous rales; no vesicular murmur. Tracheotomy was decided upon, and during the operation, when the rounded end of a hair pin was inserted to widen the opening for the introduction of the tube, the membrane shown was ejected. Next morning the child breathed easier, the lungs were clear, and the general condition was improved and continued to improve until the fourth day, when the patient died from uræmic convulsions. A brother of the child and her parents suffered from diphtheria; albumen found in their urine; they recovered. The treatment in all cases consisted of inhalations; the temperature kept at 80—85 and moist, and the administration of iron and quinine internally. The glands were enlarged in the other patients, but not in the girl.

Dr. Wm. Porter thought that the membrane had been loosened before the operation, for it is well known that the vapor from slacked lime or steam softens the membrane, that it thus becomes loosened and obstructs the trachea. The fact that the child died after the removal of the membrane, proves that the disease was true diphtheria; had it been membranous croup, recovery would probably have taken place.

Dr. A. Green—Two weeks ago I was called to see a child about two years of age, sick with croup. On examining the child I found the soft palate, the uvula, tonsils and posterior wall of the pharynx as far as I could see, covered with that peculiar kind of exudation, the so-called diphtheritic. As the false membrane extended into the larynx and trachea also, as was plainly seen by the dangerous symptoms peculiar to membranous croup, so this was the case of croup with diphtheria, a croup of the larynx and trachea as well as a croup of the pharynx, the so-called diphtheritis. But which was the primary affection? It is clear that it was either a case of primary croup of the larynx extending into the pharynx, or a primary case of dyphtheritis extending into the larynx. Having had in the last few months several cases of diphtheria, among adults and children, a few of them true diphtheritis, and none at this time of real croup under treatment, I decided that it was a primary case of diphtheria which extended into the larynx and became there a case of membranous croup. The interesting part of this case is, that it serves to illustrate a pathologico-anatomical fact, namely, that one and the same pathological process may become modified by the part upon which the morbid process is located.

The false membrane of the larynx was thrown off spontaneously without the administration of an emetic, the diphtheritic membrane—a name misapplied, because, in true diphtheria the so called membrane is the necrosed sub-epithelial connective tissue, respectively the whole mucosa with excessive infiltration of cells and low organisms, which can undergo no other change but putrefaction and decomposition. It remained there for days, and some isolated spots especially, on the right tonsil, nearly from twelve to thirteen days, before it disappeared. Again, the false membrane in the larynx was formed anew as soon as thrown off, for several nights in succession—the whole croupous process in the larynx was entirely over in about the fifteenth day of the disase—on the contrary, on those parts of the posterior wall of the parynx and fauces which cleared up the so called diphtheritic membrane did not reappear, but the parts remained clean.

After the croupous process with its threatening suffocation was entirely over, the posterior nares were lined for a few nights in succession, with a false membrane which annoyed the child much, and after snuffing, coughing, and exertions of the child to breath through the nose, it loosened and was thrown off to reappear next night.

I will only call attention here to the histological fact, that only that part of the posterior wall of the pharynx which lies opposite the nose, bears a ciliated epithelium, and consequently the morbid process behaved like croup and not like in the other parts of the pharynx which are covered by a laminated pavement epithelium.

As I considered the case to be one of a primary diphtheritic process, I abstained from topical applications for the following reasons: the so-called diphtheritis, may be only a croupous process, that is a superficial one, only a little modified by the parts affected, or a true diphtheritic process; necrosis of the whole mucosa with consequent loss of substance, or one between the two. In the first case, the diphtheritic or rather croupous membrane, if left alone will be thrown off spontaneously, and will not be reproduced in the same place, whereas if removed by force so that the part bleeds it will be reproduced on the same place, in the latter, although the necrosed part is surrounded by a sharp line of demarkation. If it be connected with the living tissue by connective tissue fibres, nerves, bloodvessels and elastic fibres, which stretch over like bridges from the living to the dead part, these bridges must first be separated by a reactive inflammation with suppuration, before the slough can become separated. If topical applications, I mean those severe mechanical ones, as the probang or solid nitrate of silver, etc., to loosen and remove these sloughs, are used, we may favor the absorption of septic matter, by destroying the granulations, the protective wall against absorption, and produce septicæmia, which is the causa mortis of most of those that die from diphtheria. I will add here that the child got entirely well.

*Resection of the Knee Joint.*—Dr. Gregory showed portions of bone removed during resection of the knee joint. The patient was 20 years of age; no history of injury; the disease had run the usual course of white swelling for eight months. The operation consisted in making a long horseshoe shaped anterior flap, then about an inch and a half of the lower end of the femur and the patella, and subsequently three more layers of bone were removed. When the last piece was taken away a sequestrum three inches long was discovered in the canal of the femur, evidently the result of central necrosis. After taking a layer from the tibia the bones were adjusted and a McEntire splint applied. The limb is three inches shorter than the other. Patient is doing well.

Dr. Spencer read a paper on amyl nitrite in diseases of the ear. Published in the June number of this journal.

At this meeting of the Society, the report of the committee which had been appointed at a previous meeting to consider the feasibility of employing a stenographer to report the proceedings of the Society, was adopted and, on the recommendation of the committee, Dr. R. M. Funkhouser was engaged to fill this office.—*Hospital Reports.*

